# The role and mechanism of esketamine in preventing and treating remifentanil-induced hyperalgesia based on the NMDA receptor–CaMKII pathway

**DOI:** 10.1515/biol-2022-0816

**Published:** 2024-01-31

**Authors:** Jiafang Wang, Yankun Feng, Zhong Qi, Jin Li, Zhijun Chen, Jinming Zhang, Degang Zhu

**Affiliations:** Department of Anesthesiology, Wuhan No. 1 Hospital, No. 215 Zhongshan Avenue, Qiaokou District, Wuhan, Hubei, 430022, China; Hubei Provincial Key Laboratory for Applied Toxicology, Hubei Center for Disease Control and Prevention, No. 35 Zhuodaoquan North Road, Hongshan District, Wuhan 430079, China

**Keywords:** remifentanil, esketamine, pain management, CaMKⅡ, GluN2B, opioids

## Abstract

Remifentanil-induced hyperalgesia (RIH) is a common clinical phenomenon that limits the use of opioids in pain management. Esketamine, a non-competitive *N*-methyl-d-aspartate (NMDA) receptor antagonist, has been shown to prevent and treat RIH. However, the underlying effect mechanism of esketamine on RIH remains unclear. This study aimed to investigate the role and mechanism of esketamine in preventing and treating RIH based on the NMDA receptor–CaMKIIα pathway. In this study, an experimental animal model was used to determine the therapeutic effect of esketamine on pain elimination. Moreover, the mRNA transcription and protein expression levels of CaMKII and GluN2B were investigated to offer evidence of the protective capability of esketamine in ameliorating RIH. The results demonstrated that esketamine attenuated RIH by inhibiting CaMKII phosphorylation and downstream signaling pathways mediated by the NMDA receptor. Furthermore, ketamine reversed the upregulation of spinal CaMKII induced by remifentanil. These findings suggest that the NMDA receptor–CaMKII pathway plays a critical role in the development of RIH, and ketamine’s effect on this pathway may provide a new therapeutic approach for the prevention and treatment of RIH.

## Graphical abstract

**Figure:**
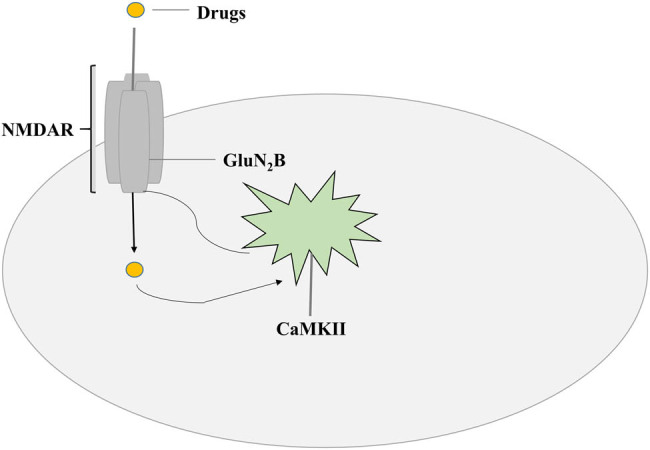

## 1 Introduction

Remifentanil is a potent, short-acting synthetic opioid that has gained widespread use in clinical settings as an analgesic agent. It was first approved by the US Food and Drug Administration in 1996 and has since become a popular choice for use during anesthesia and in acute pain management [1,2]. Remifentanil works by binding to μ-opioid receptors in the central nervous system, resulting in potent analgesic effects [3]. It has a rapid onset of action and a short duration of effect, which makes it particularly useful in situations where precise control of the depth and duration of anesthesia is required [4]. Unlike other opioids, remifentanil is rapidly metabolized by plasma and tissue esterase, resulting in a rapid offset of its effects [5]. This unique property makes it particularly useful in situations where a rapid recovery from anesthesia is desired, such as in outpatient surgical procedures. Although remifentanil has several advantages in clinical settings, the use of remifentanil is not without potential drawbacks. Like other opioids, remifentanil has a high potential for addiction, and its use can lead to physical dependence and withdrawal symptoms if not used appropriately [6]. Therefore, it is essential to use remifentanil only under supervision and in the appropriate clinical context.

Esketamine is a novel medication that has recently gained attention as a potential treatment for major depressive disorder and treatment-resistant depression (TRD) [7,8,9]. It is a derivative of ketamine, a dissociative anesthetic that has been used clinically for several decades. Esketamine is a non-competitive *N*-methyl-d-aspartate (NMDA) receptor antagonist that works by rapidly increasing glutamate transmission in the brain [10,11]. This increased glutamate signaling has been linked to the rapid and sustained antidepressant effects of esketamine [12]. Esketamine has been shown to have rapid and robust antidepressant effects, with some patients reporting improvement within hours of treatment. However, its use is not without controversy, as it has potential side effects, including dissociation, dizziness, and nausea [10,13]. Despite the potential risks, esketamine represents a promising new treatment option for patients with TRD who have not responded to traditional antidepressant therapies [14].

Remifentanil and esketamine are two powerful drugs used in anesthesia and pain management [15]. While they have different mechanisms of action, when used together, they can offer several advantages, such as reduced opioid requirements and improved patient satisfaction [16]. Remifentanil is an opioid that provides potent analgesia, but it can also cause respiratory depression and other side effects. When used in combination with esketamine, the amount of remifentanil required to achieve the desired level of analgesia can be reduced, thereby reducing the risk of opioid-related side effects [17]. Furthermore, synergistic effects would be able to be counted from the combination of remifentanil and esketamine. As a potent NMDA receptor antagonist, esketamine can enhance the analgesic effects of opioids, leading to greater pain relief than either drug alone.

While the combination of remifentanil and esketamine can offer several advantages in terms of anesthesia and pain management, there are still some unknown factors that need to be considered. In this study, the effect of the combined treatment of remifentanil and esketamine on experimental animals on the scale of reducing pain and improving pain tolerance was investigated to offer ideas for figuring out the question mentioned above.

## 2 Materials and methods

### 2.1 Materials

Remifentanil was purchased from the Yichang Renfu Pharmaceutical Industry. Esketamine was bought from Jiangsu Hengrui Medicine. *N*-Methyl-d-aspartic acid (NMDA) was purchased from Selleckchem. Polyvinylidene fluoride (PVDF) membrane was bought from Millipore. Antibodies used in this study were purchased from Abcam (CaMKII and p-CaMKII) and Cell Signaling Technology (GluN2B and GAPDH). All other chemicals were purchased from Sigma-Aldrich.

### 2.2 Experimental animal

Male ICR mice aged 10–12 weeks and weighing 30–35 g were bought from the Hubei Provincial Laboratory Animal Public Service Center. Mice were kept in separate cages with an appropriate density before the formal experiment to adapt to the environment. The environment of the animal facility was controlled as 12 h of light and 12 h of darkness with a room temperature of 20–23°C. Mice were free to access food and water except 12 h before the operation.

A total of 60 mice were randomly separated to 6 groups (*n* = 10), which were labeled as the control group, sham control group (called “P” in the figure legend), NMDA agonist group (called “NMDA” in the figure legend), remifentanil group (called “R” in the figure legend), remifentanil combined with esketamine group (called “RE” in the figure legend), and remifentanil combined with esketamine and intrathecal NMDA agonist group (called “REN” in the figure legend). Mice were subjected to a procedure in which incision was made in the left hind foot with inhalation of 1–2% sevoflurane for anaesthetization except for controls. For the sham control group, there was no further treatment before tests; for the NMDA agonist group, 5 μL of *N*-methyl-d-aspartic acid (5 nM) was intrathecally injected half an hour before surgery; for modeling of the remifentanil group, continuous subcutaneous pumping of remifentanil (80 μg/kg) was applied to mice; for the remifentanil combined with esketamine group, continuous subcutaneous pumping of remifentanil (80 μg/kg) and ketamine (administered intravenously by drip, 1 mg/kg) was used. For the remifentanil combined with esketamine and intrathecal NMDA agonist group, remifentanil (80 μg/kg) and ketamine (1 mg/kg) were used half an hour after intrathecal injection of *N*-methyl-d-aspartic acid (5 nM). This study was approved by Wuhan No.1 Hospital’s Institutional Animal Care and Use Committee (permit number: IACUC-202120155) in compliance with institutional guidelines for the care and use of animals.


**Ethical approval:** The research related to animal use has been complied with all the relevant national regulations and institutional policies for the care and use of animals and has been approved was approved by Wuhan No. 1 Hospital s Institutional Animal Care and Use Committee (permit number: IACUC-202120155).

### 2.3 Hot plate test

First, the plate was preheated, and the thermostatic water bath was adjusted to 55 ± 0.5°C, then the mice were put into the beaker, and the timing was started from the time the mice landed on the hind limbs and left the ground, as well as the phenomenon of licking the hind feet on the surgical side. A timer was used to start the timing until the mice licked their hind feet. In order not to burn the mice, each test did not exceed 30 s. The result would be recorded as 30 s if the mice did not lick their hind feet for more than 30 s. Each mouse was measured twice in a row, and each test was separated by 5 min, and the average result of the two tests was taken as the thermal pain threshold of the mouse.

### 2.4 Paw withdrawal test

Mice were placed in a resin cylinder with a diameter of 9 cm and a height of 15 cm with a wire mesh at the bottom, in which the mice could move freely. The mice were stimulated on the sole of the foot with von Frey filaments (folding force ranging from 0.008 to 2 g). The stimulation interval was at least 1 min. The mechanical pain threshold was tested by a modified up-down method. Stimulation was initiated by a fibrous wire with a folding force of 0.4 g. Regardless of whether the mice stimulated by the fiber filament of the folded force had a positive response, each strength was repeatedly stimulated five times, and when the stimulation at the folded force showed at least three positive responses, the strength was recorded as the upper limit strength, and the positive response rate of the strength (number of positive responses/5 × 100%) was the upper limit strength positive rate, and if the positive response was <3 times, the strength was adjusted upward by one level for determination; when the strength inducing a negative response positive reaction <3 times after five times of repeated testing, the strength was recorded as the lower limit strength, and the positive reaction rate of this strength was the lower limit strength positive rate; similarly, if the strength induced positive reaction ≥3 times, the strength was adjusted downward by one level for determination.

### 2.5 Real-time quantitative PCR

Primary mouse sensory cortical (S1) tissue (10 mg) was isolated by surgical instrumentation, and 300 μL of Trizol was added to lyse the tissue on ice for 5 min. Subsequently, the tissue was carefully homogenized using an electric tissue grinder. After homogenization of the tissue, Trizol was added to make up a total volume of 1 mL, followed by the addition of 200 μL of trichloromethane to vortex the tube vigorously to mix the liquid completely. Centrifuge the mixture at 12,000 rpm for 15 min to separate the liquid phases. Carefully aspirate the clarified solution into a clean centrifuge tube, add 500 μL of isopropanol, and mix the liquid by inverting up and down. After careful removal of the supernatant, add 1 mL of precooled 75% ethanol solution and wash the RNA by inverting up and down. Centrifuge at 7,500 rpm for 5 min at room temperature to precipitate the RNA, and open the lid of the tube to dry the RNA after complete removal of the supernatant. The protocol was performed as previously described [18].

The collected RNA was reverse transcribed to cDNA, and gene expression was detected using a qPCR instrument. qPCR reaction conditions were set to 10 min at 95°C for the DNA deconvolution phase, 15 s at 95°C and 30 s at 60°C for the PCR phase, 40 cycles, and 15 s at 95°C followed by 1 min at 60°C for the melting curve phase and 15 s at 95°C for the final reaction. Three replicates were set for each experimental group, and the Ct values collected from each well were analyzed to show the relative expression of each gene (CaMKII, Grin2b) using the housekeeping gene GAPDH as the reference. The primers were designed by authors through the websites (https://www.ncbi.nlm.nih.gov/ and https://pga.mgh.harvard.edu/primerbank/) and synthesized by Sangon Biotech (Shanghai, China). The primer sequences were as follows: CaMKII forward primer TGCCTGGTGTTGCTAACCC reverse primer CCATTAACTGAACGCTGGAACT; Grin2b forward primer GCCATGAACGAGACTGACCC reverse primer GCTTCCTGGTCCGTGTCATC; GAPDH forward primer AGGTCGGTGTGAACGGATTTG reverse primer TGTAGACCATGTAGTTGAGGTCA.

### 2.6 Western blotting

S1 tissues from various groups were collected after treatments, and then they were homogenized with RIPA lysis buffer by tissue grinder. Lysates were transferred to clean tubes for centrifugation with 14,000 rpm at 4°C for 10 min, and the supernatant was collected. Afterward, the BCA kit (Beyotime, China) was used to calculate the protein concentration of samples to normalize the amount of protein. The 40 μg protein was added to the lanes and was subjected to SDS–PAGE and then transferred to PVDF membrane by using a semi-dry transfer system (Bio-Rad, USA). About 5% skim milk was used to block the membrane for 1 h at room temperature. PVDF membrane was soaked at diluted primary antibody overnight at 4 °C, and secondary antibody was used to incubate with membrane for 1 h at room temperature after an appropriate membrane-washing procedure. The visualization of corresponding proteins was achieved by using an ECL chemiluminescence detection system, and values of fluorescence were analyzed by ImageJ software. The protocol was performed as previously described [19].

### 2.7 Statistics

Reported data from this article were collected from three independent samples, except a certain number of samples were mentioned, and data were expressed as mean ± standard deviations (SD). Differences were identified using a Student’s *t*-test performed by GraphPad Prism 7 (GraphPad Software, USA) and were considered significant when *p* < 0.05.

## 3 Results

### 3.1 Remifentanil combined with esketamine reduces pain sensation in experimental animals

**Figure 1:**
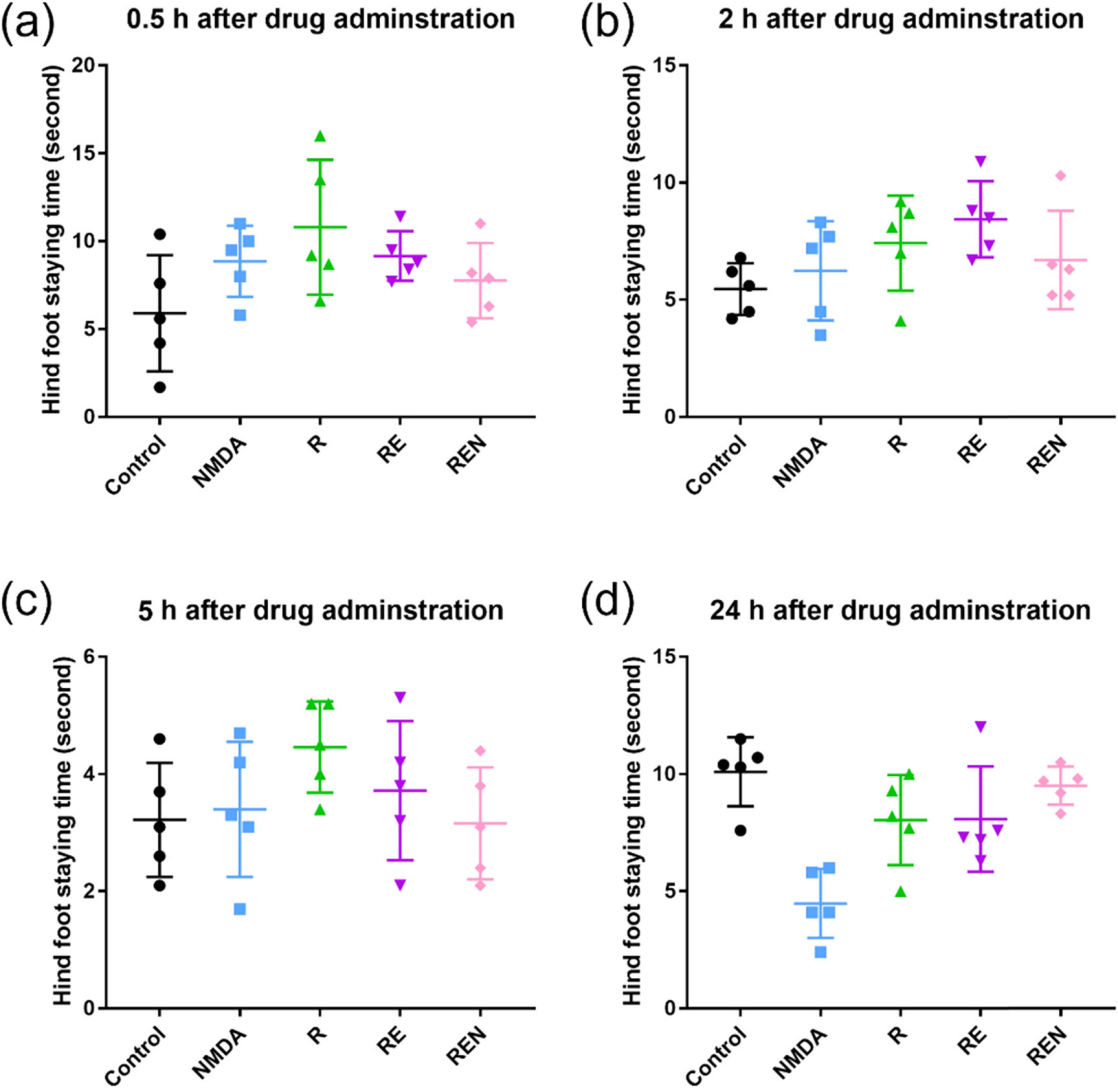
Hindfoot staying time of experimental animal with various treatments after drug administration. (a) 0.5 h after drug administration. (b) 2 h after drug administration. (c) 5 h after drug administration. (d) 24 h after drug administration. Data are expressed as means ± SD (*n* = 6).

In the 0.5-h experiment, the control group exhibited a hind paw withdrawal latency of 5.9 ± 2.9 s, while the group treated with NMDA agonist showed a hind paw withdrawal time of 8.9 ± 1.8 s (Figure 1a). Remifentanil, despite its shorter half-life, exhibited pain inhibition in mice 0.5 h after administration, with a hind paw withdrawal time of 10.8 ± 3.4 s. The combination group of remifentanil and esketamine showed a shorter pain perception time compared to the remifentanil group, with a time of 9.2 ± 1.3 s. However, when NMDA agonist was applied to the combination group of remifentanil and esketamine, the mice’s pain tolerance time was further shortened to 7.8 ± 1.9 s, demonstrating the synergistic effect of the two drugs. In the study of drug cessation for 2 h, the control group showed no significant difference with a hind paw withdrawal time of 5.5 ± 1.0 s, whereas the NMDA agonist group had a withdrawal time of 6.2 ± 1.9 s (Figure 1b). As the drug was metabolized and eliminated, the remifentanil group showed a hind paw withdrawal time of 7.4 ± 1.8 s, whereas the combination group of remifentanil and esketamine had a time of 8.4 ± 1.4 s, with no significant difference compared to the analgesic effect 0.5 h after drug cessation. In the 5-h cessation experiment, all experimental groups of mice exhibited pain intolerance: control group, 3.2 ± 0.9 s; NMDA agonist group, 3.4 ± 1.0 s; remifentanil group, 4.5 ± 0.7 s; combination group of remifentanil and esketamine, 3.7 ± 1.1 s; and REN group, 3.2 ± 0.9 s (Figure 1c). This indicates that the anesthesia effect gradually weakened and disappeared as drug cessation time increased. After 24 h of cessation, the control group had a latency of 10.1 ± 1.1 s, whereas the NMDA agonist group had a latency of 4.5 ± 1.3 s (Figure 1d). This result may be related to the sustained NMDA activation and pain sensation resulting from the administration method. The remifentanil group and the drug combination group had similar performances, with hind paw withdrawal times of 8.0 ± 1.7 s and 8.1 ± 2.0 s, respectively, whereas the REN group had a time of 9.5 ± 0.3 s.

### 3.2 Remifentanil combined with esketamine improves the tolerance of experimental animals to mechanical pain

**Figure 2:**
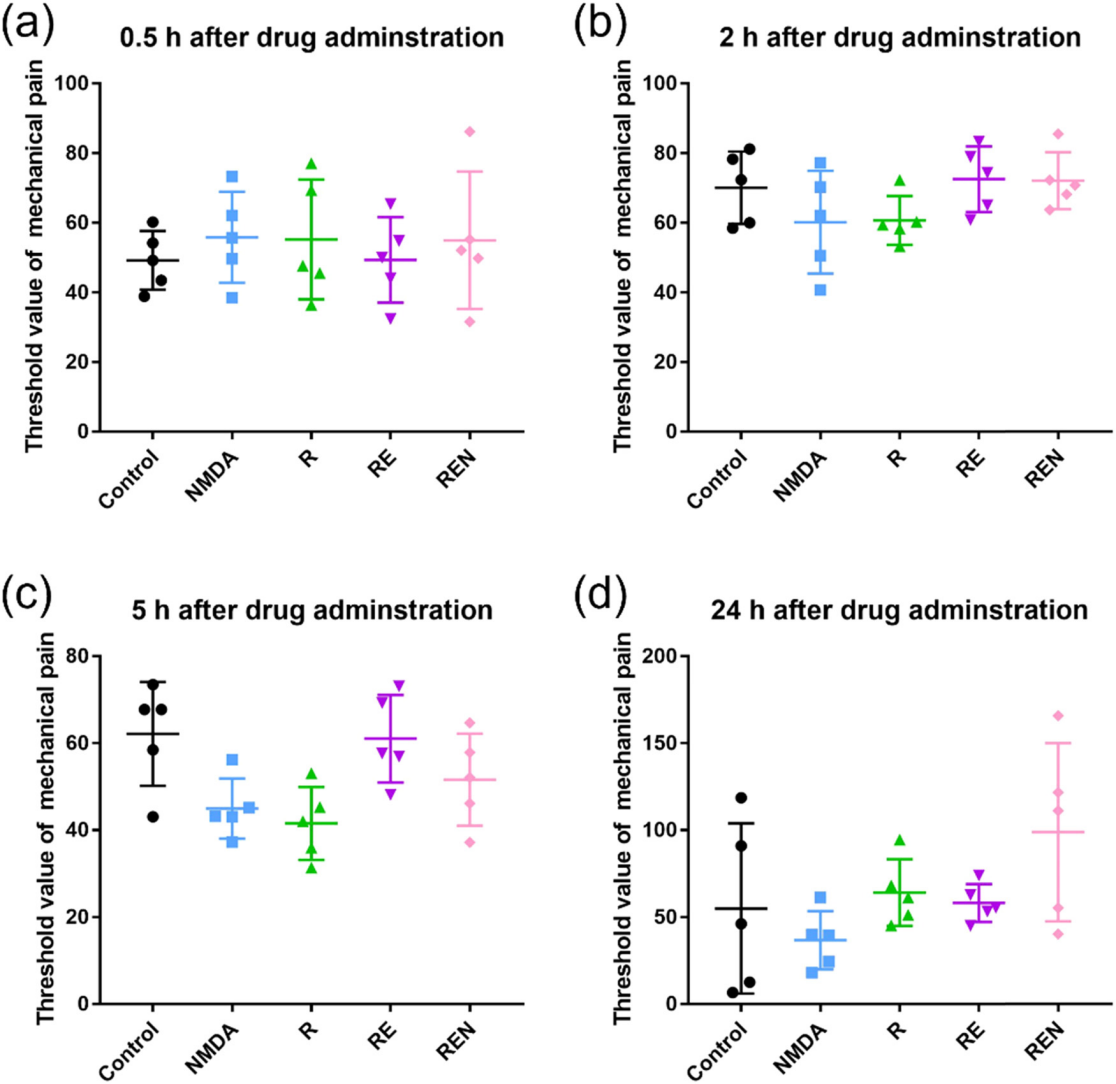
Threshold values of experimental animals with various treatments after drug administration. (a) 0.5 h after drug administration. (b) 2 h after drug administration. (c) 5 h after drug administration. (d) 24 h after drug administration. Data are expressed as mean ± SD (*n* = 6).

In the experiment conducted 0.5 h after drug cessation, the pain threshold of the control group was 49.2 ± 7.5, while that of the NMDA agonist-treated group of mice was 55.9 ± 11.7. Remifentanil did not show inhibition of mechanical pain after 0.5 h of drug cessation, and its pain threshold was 55.2 ± 15.4. In the group treated with a combination of remifentanil and esketamine, there was no significant difference in pain threshold compared to the remifentanil group, which was 49.3 ± 10.0. The application of NMDA agonist in the group treated with a combination of remifentanil and esketamine did not alter the sensitivity to mechanical pain, with a pain threshold of 55.0 ± 17.6 (Figure 2a). In the study conducted 2 h after drug cessation, the pain threshold of each group was elevated compared to that of the 0.5-h drug cessation group. The control group had a threshold of 70.0 ± 9.3, the NMDA agonist group was 60.2 ± 13.2, the remifentanil group was 60.7 ± 6.3, the combination group of remifentanil and es-ketamine was 72.6 ± 8.4, and the combination group of NMDA agonist, remifentanil, and esketamine was 72.2 ± 7.3 (Figure 2b). Interestingly, in the experiment conducted 5 h after drug cessation, the control group maintained a higher pain threshold of 62.1 ± 10.7. The NMDA agonist group and the remifentanil group both exhibited intolerance to mechanical pain, with values of 45.0 ± 6.2 and 41.6 ± 7.5, respectively (Figure 2c). Compared to remifentanil alone, the combination of remifentanil and esketamine better maintained a high pain threshold, demonstrating that the synergistic effect of remifentanil and esketamine was better than the analgesic effect of each drug alone. In the experiment conducted 24 h after drug cessation, the pain threshold of the control group was 55.0 ± 43.7, which may be due to individual differences in pain sensitivity (Figure 2d). The NMDA agonist group had a threshold of 36.8 ± 15.0, the remifentanil group had a threshold of 64.1 ± 17.1, and the combination group of remifentanil and esketamine had a threshold of 58.2 ± 9.7. Interestingly, the pain threshold of the group treated with a combination of NMDA agonist, remifentanil, and esketamine was higher, at 98.8 ± 45.8, which may be related to individual differences in the experimental animals.

### 3.3 Effects of remifentanil combined with esketamine on the expression of CaMKII and Grin2b in sensory cortical area

**Figure 3:**
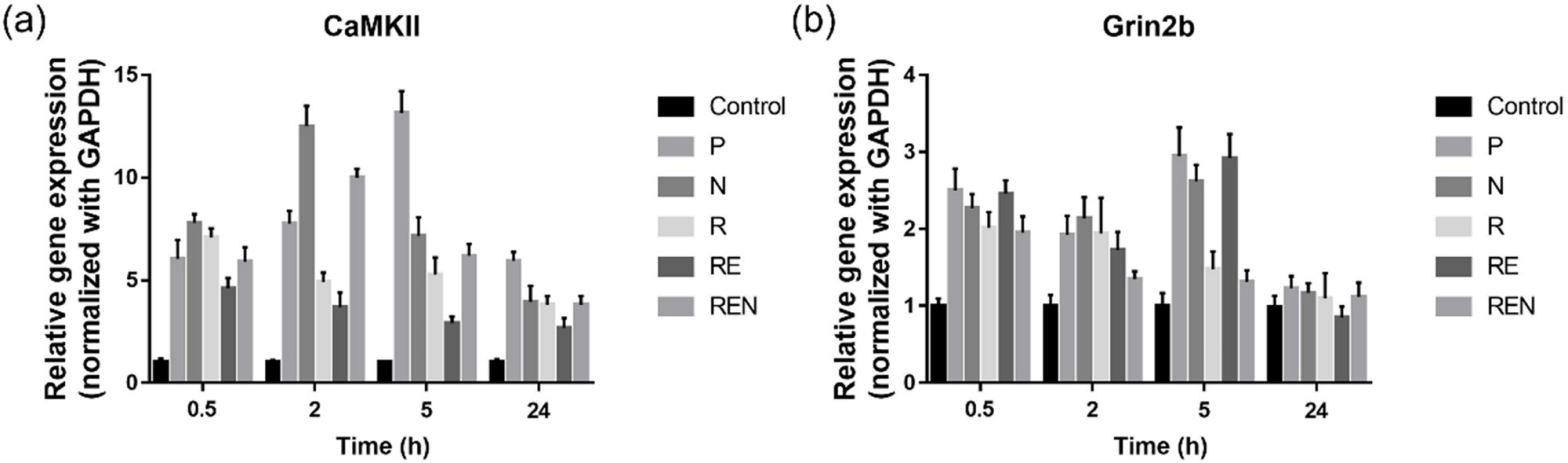
Effects of remifentanil combined with esketamine on the expression of CaMKII and Grin2b in sensory cortical area. (a) The expression of CaMKII in different groups from 0.5 h to 24 h. (b) The expression of Grin2b in different groups from 0.5h to 24 h.

The qPCR was employed to investigate the effects of remifentanil combined with esketamine on the expression of CaMKII and Grin2b in the sensory cortical area at various timepoints (Figure 3). After 0.5 h of drug cessation, mRNA transcription of CaMKII and Grin2b in the sensory cortex showed significant upregulation in all treatment groups compared to the control group. After 2 h of drug cessation, the expression level of CaMKII in the NMDA agonist treatment group remained high, but the remifentanil group and the remifentanil combined with esketamine group showed downregulation compared to the NMDA agonist treatment group despite being higher than the control group. The expression pattern of Grin2b was different from CaMKII, showing relatively high expression in the P group, NMDA agonist treatment group, and remifentanil group, while the remifentanil combined with esketamine group showed downregulation of Grin2b expression compared to the remifentanil treatment group. After 5 h of drug cessation, the relative expression levels of CaMKII and Grin2b in the P group were the highest among all groups, while the CaMKII expression level in the remifentanil combined with esketamine group was the lowest except for the control group, but with a higher level of Grin2b expression. After 24 h of drug cessation, the CaMKII expression pattern in each group was similar to that at 5 h of withdrawal, while the expression of Grin2b in each group was no longer significantly different from that in the control group.

### 3.4 Effects of remifentanil combined with esketamine on the protein regulation of CaMKII and GluN2B in sensory cortical area

**Figure 4:**
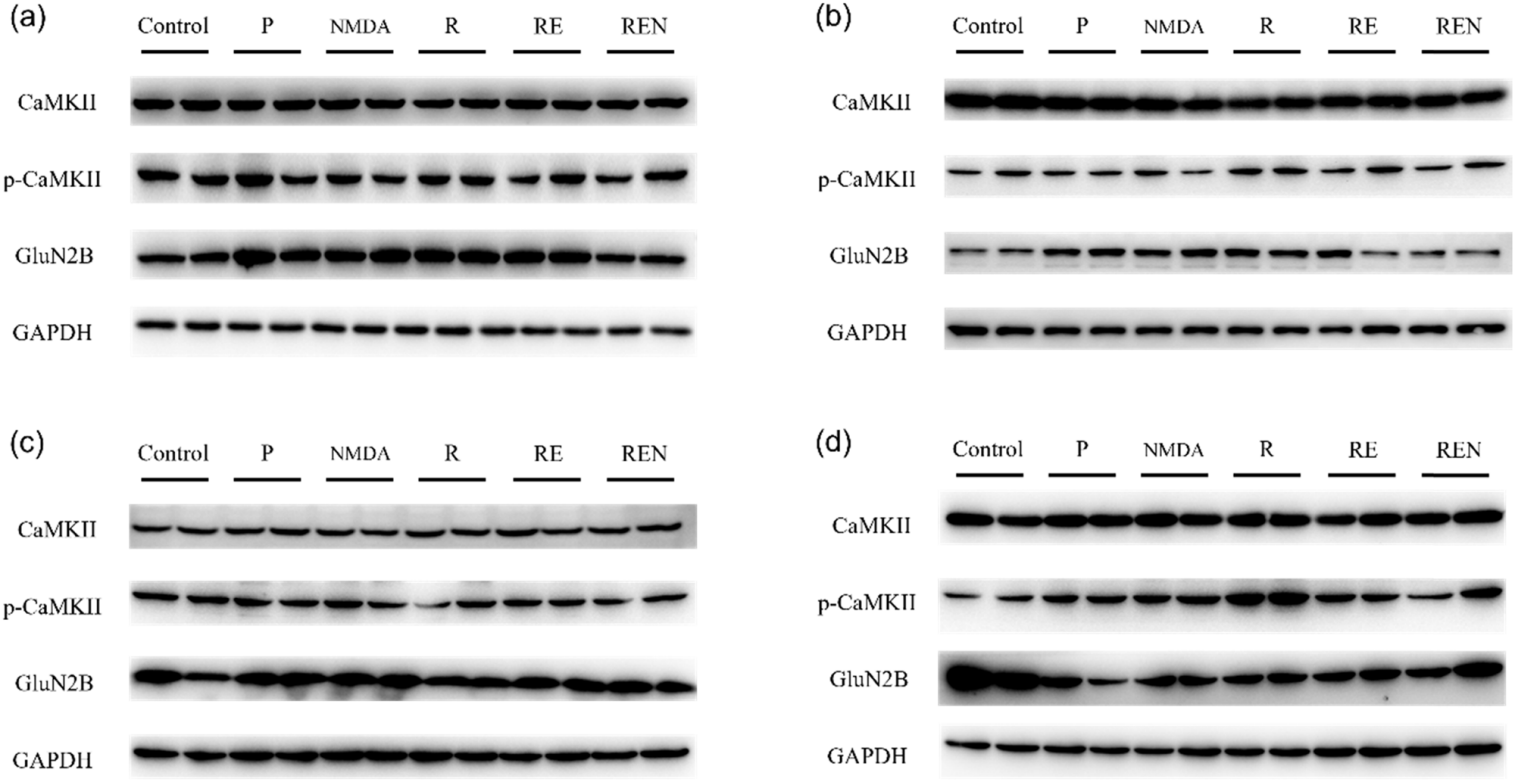
Effects of remifentanil combined with esketamine on the protein regulation of CaMKII and GluN2B in various timepoints post medication. (a) 0.5 h after drug administration. (b) 2 h after drug administration. (c) 5 h after drug administration. (d) 24 h after drug administration. (*n* = 2).

The immunoblot was used to determine the protein expression in the sensory cortical area (Figure 4). After 0.5 h of drug cessation, there was no significant increase in the expression of phosphorylated CaMKII in all treatment groups, which may be related to residual anesthesia effects. However, the expression of the NMDA receptor subunit GluN2B was lower in the control group but was upregulated in the sham surgery group, NMDA treatment group, remifentanil group, and remifentanil combined esketamine group. The expression of GluN2B in the remifentanil plus esketamine group treated with NMDA was similar to that in the control group. After 2 h of drug cessation, an upregulation in phosphorylated CaMKII expression was observed only in the remifentanil group. Compared to the result from 0.5-h drug cessation, the expression pattern of GluN2B was different, with lower expression in the control group, remifentanil plus esketamine group treated with NMDA, and remifentanil group, and increased expression in other treatment groups relative to the control group. In the experiment after 5 h of drug cessation, the expression of phosphorylated CaMKII was downregulated in the remifentanil group, remifentanil plus esketamine group treated with NMDA, and remifentanil group, compared to the control group. However, for the expression of GluN2B, all treatment groups except the remifentanil group showed an upregulation compared to the control group. After 24 h of drug cessation, there was a significant upregulation in the expression of phosphorylated CaMKII in the remifentanil group, possibly due to remifentanil-induced hyperalgesia (RIH). The expression of GluN2B in all treatment groups was downregulated compared to the control group.

## 4 Discussion

In this study, we demonstrated that esketamine attenuates RIH based on the NMDA receptor CaMKII pathway.

Remifentanil and esketamine are two medications commonly used in anesthesia and pain management [20,21]. Remifentanil is a potent opioid analgesic, while esketamine, a derivative of ketamine, is a dissociative anesthetic that acts on the *N*-methyl-d-aspartate (NMDA) receptor [22].

There is evidence to suggest that the combination of remifentanil and esketamine may have a synergistic effect in reducing pain and improving anesthesia [23]. CaMKII is a multifunctional enzyme that plays an important role in synaptic plasticity and learning and memory [24]. It is activated by calcium influx into cells and is involved in a variety of cellular processes, including gene expression, ion channel regulation, and neurotransmitter release [25]. Recent studies have suggested that CaMKII may also play a role in pain modulation and the effects of opioids and NMDA receptor antagonists.

Previous studies investigated the effects of combined remifentanil and esketamine on CaMKII activation in a rat model of neuropathic pain [26,27]. The results showed that the combination treatment led to a significant decrease in CaMKII activation in the spinal cord dorsal horn, which is a key region involved in pain processing [28,29]. This suggests that the combination treatment may have a potential therapeutic effect in treating neuropathic pain by modulating CaMKII activity. Another study investigated the effects of combined remifentanil and esketamine on CaMKII activation in the hippocampus, a brain region involved in learning and memory [30]. The results showed that the combination treatment led to a significant increase in CaMKII activation in the hippocampus, which suggests that the combination treatment may have a potential therapeutic effect in improving cognitive function. While these studies suggest that the combination of remifentanil and esketamine may have potential therapeutic effects on CaMKII activity, more research is needed to fully understand the mechanisms involved and to determine the clinical implications of these findings [2,31]. Additionally, further studies are needed to investigate the potential side effects of this combination treatment, as both remifentanil and esketamine can have adverse effects on the cardiovascular and respiratory systems.

Nerve cell damage is closely related to brain function. It has been reported that ketamine administration can induce neuroapoptosis in primary cultured cortical neurons [32,33]. For esketamine, it increases cell viability in LPS-induced primary cultured astrocytes and ameliorates the pyroptosis of astrocytes induced by LPS exposure by inhibiting the expression levels of cl-caspase-1 and IL-18 [34]. Esketamine can also alleviate apoptosis through the restoration of mitochondrial function in cortical neuronal cells induced by H_2_O_2_ [35]. For remifentanil, it reduces glutamate toxicity and increases cell viability in cultured neurons from the rat olfactory bulb [36]. Moreover, remifentanil substantially decreases neuronal apoptosis levels and increases the B-cell lymphoma 2 (Bcl-2)/Bcl-2-associated X protein (Bax) ratio in cerebral ischemia-reperfusion injury rats [37].

However, there are also some limitations in this study. First, we have not performed an *in vitro* experiment to explore the effect of esketamine on RIH. Second, we have not explored the effects of remifentanil, esketamine, and ketamine on apoptosis and necrosis of nerve cells. In future, we would like to further explore the effect of esketamine on the NMDA receptor–CaMKIIα pathway in RIH mice by gene overexpression and siRNA technology. Moreover, lactate dehydrogenase assay is also needed to explore the apoptotic processes and the possibility of toxicity of those drugs.

## 5 Conclusion

In conclusion, while there is evidence to suggest that the combination of remifentanil and esketamine may have potential therapeutic effects on CaMKII activity, further research is needed to fully understand the mechanisms involved and to determine the clinical implications of these findings. Additionally, the potential side effects of this combination treatment should be carefully considered before it is used in clinical practice.

## References

[1] Grape S, Kirkham KR, Frauenknecht J, Albrecht E. Intra-operative analgesia with remifentanil vs. dexmedetomidine: a systematic review and meta-analysis with trial sequential analysis. Anaesthesia. 2019;74(6):793–800. 10.1111/anae.14657 30950522

[2] Eleveld DJ, Colin P, Absalom AR, Struys MMRF. Target-controlled-infusion models for remifentanil dosing consistent with approved recommendations. Br J Anaesth. 2020;125(4):483–91. 10.1016/j.bja.2020.05.051 32654750

[3] Hughes LM, Irwin MG, Nestor CC. Alternatives to remifentanil for the analgesic component of total intravenous anaesthesia: a narrative review. Anaesthesia. 2023;78(5):620–5. 10.1111/anae.15952 36562193

[4] Birgenheier NM, Stuart AR, Egan TD. Soft drugs in anesthesia: remifentanil as prototype to modern anesthetic drug development. Curr Opin Anaesthesiol. 2020;33(4):499–505. 10.1097/ACO.0000000000000879 32530892

[5] Yi S, Cao H, Zheng W, Wang Y, Li P, Wang S, et al. Targeting the opioid remifentanil: Protective effects and molecular mechanisms against organ ischemia-reperfusion injury. Biomed Pharmacother. 2023;167:115472. 10.1016/j.biopha.2023.115472 37716122

[6] Karila L, Marillier M, Chaumette B, Billieux J, Franchitto N, Benyamina A. New synthetic opioids: Part of a new addiction landscape. Neurosci Biobehav Rev. 2019;106:133–40. 10.1016/j.neubiorev.2018.06.010 30217656

[7] Terao I, Tsuge T, Endo K, Kodama W. Comparative efficacy, tolerability and acceptability of intravenous racemic ketamine with intranasal esketamine, aripiprazole and lithium as augmentative treatments for treatment-resistant unipolar depression: A systematic review and network meta-analysis. J Affect Disord. 2023;346:49–56. 10.1016/j.jad.2023.11.023 37949235

[8] Bahji A, Vazquez GH, Zarate CA Jr. Comparative efficacy of racemic ketamine and esketamine for depression: A systematic review and meta-analysis. J Affect Disord. 2021;278:542–55. 10.1016/j.jad.2020.09.071 PMC7704936 33022440

[9] K Freind JM, Beserra FR, Menezes BS, Mograbi DC. Therapeutic protocols using ketamine and esketamine for depressive disorders: A systematic review. J Psychoact Drugs. 2023;28:1–17. 10.1080/02791072.2023.2248989 37638529

[10] Henter ID, Park LT, Zarate CA Jr. Novel glutamatergic modulators for the treatment of mood disorders: current status. CNS Drugs. 2021;35(5):527–43. 10.1007/s40263-021-00816-x PMC8201267 33904154

[11] Levinta A, Meshkat S, McIntyre RS, Ho C, Lui LMW, Lee Y, et al. The association between stage of treatment-resistant depression and clinical utility of ketamine/esketamine: A systematic review. J Affect Disord. 2022;318:139–49. 10.1016/j.jad.2022.08.050 36049604

[12] Daly EJ, Singh JB, Fedgchin M, Cooper K, Lim P, Shelton RC, et al. Efficacy and safety of intranasal esketamine adjunctive to oral antidepressant therapy in treatment-resistant depression: a randomized clinical trial. JAMA Psychiatry. 2018;75(2):139–48. 10.1001/jamapsychiatry.2017.3739 PMC5838571 29282469

[13] Ceban F, Rosenblat JD, Kratiuk K, Lee Y, Rodrigues NB, Gill H, et al. Prevention and management of common adverse effects of ketamine and esketamine in patients with mood disorders. CNS Drugs. 2021;35(9):925–34. 10.1007/s40263-021-00846-5 34363603

[14] Kaur U, Pathak BK, Singh A, Chakrabarti SS. Esketamine: a glimmer of hope in treatment-resistant depression. Eur Arch Psychiatry Clin Neurosci. 2021;271(3):417–29. 10.1007/s00406-019-01084-z 31745646

[15] Nie J, Chen W, Jia Y, Zhang Y, Wang H. Comparison of remifentanil and esketamine in combination with propofol for patient sedation during fiberoptic bronchoscopy. BMC Pulm Med. 2023;23(1):254. 10.1186/s12890-023-02517-1 PMC10334652 37430293

[16] Zhong Y, Jiang M, Wang Y, Su T, Lv Y, Fan Z, et al. Evaluating efficacy and safety of sub-anesthetic dose esketamine as an adjuvant to propofol/remifentanil analgosedation and spontaneous respiration for children flexible fibreoptic bronchoscopy: a prospective, double-blinded, randomized, and placebo-controlled clinical trial. Front Pharmacol. 2023;14:1184663. 10.3389/fphar.2023.1184663 PMC10203403 37229247

[17] Su Y, Zhang J, Wang H, Gu Y, Ouyang H, Huang W. The use of Esketamine in CT-guided percutaneous liver tumor ablation reduces the consumption of remifentanil: a randomized, controlled, double-blind trial. Ann Transl Med. 2022;10(12):704. 10.21037/atm-22-2756 PMC9279786 35845514

[18] Vuletic A, Konjevic G, Milanovic D, Ruzdijic S, Jurisic V. Antiproliferative effect of 13-cis-retinoic acid is associated with granulocyte differentiation and decrease in cyclin B1 and Bcl-2 protein levels in G0/G1 arrested HL-60 cells. Pathol Oncol Res. 2010;16(3):393–401. 10.1007/s12253-009-9241-2 20084480

[19] Scherbakov AM, Vorontsova SK, Khamidullina AI, Mrdjanovic J, Andreeva OE, Bogdanov FB, et al. Novel pentacyclic derivatives and benzylidenes of the progesterone series cause anti-estrogenic and antiproliferative effects and induce apoptosis in breast cancer cells. Invest N Drugs. 2023;41(1):142–52. 10.1007/s10637-023-01332-z PMC9875769 36695998

[20] Feeney A, Papakostas GI. Pharmacotherapy: Ketamine and Esketamine. Psychiatr Clin North Am. 2023;46(2):277–90. 10.1016/j.psc.2023.02.003 37149345

[21] Kisilewicz M, Rosenberg H, Vaillancourt C. Remifentanil for procedural sedation: a systematic review of the literature. Emerg Med J. 2017;34(5):294–301. 10.1136/emermed-2016-206129 28249938

[22] Mion G, Villevieille T. Ketamine pharmacology: an update (pharmacodynamics and molecular aspects, recent findings). CNS Neurosci Ther. 2013;19(6):370–80. 10.1111/cns.12099 PMC6493357 23575437

[23] Xin N, Yan W, Jin S. Efficacy of analgesic propofol/esketamine and propofol/fentanyl for painless induced abortion: a randomized clinical trial. Biomed Res Int. 2022;2022:5095282. 10.1155/2022/5095282 PMC9203225 35722469

[24] Yasuda R, Hayashi Y, Hell JW. CaMKII: a central molecular organizer of synaptic plasticity, learning and memory. Nat Rev Neurosci. 2022;23(11):666–82. 10.1038/s41583-022-00624-2 36056211

[25] Nicoll RA, Schulman H. Synaptic memory and CaMKII. Physiol Rev. 2023;103(4):2877–2925. 10.1152/physrev.00034.2022 PMC10642921 37290118

[26] Zhou HY, Chen SR, Pan HL. Targeting N-methyl-d-aspartate receptors for treatment of neuropathic pain. Expert Rev Clin Pharmacol. 2011;4(3):379–88. 10.1586/ecp.11.17 PMC3113704 21686074

[27] Raffa RB, Pergolizzi JV Jr. Opioid-induced hyperalgesia: is it clinically relevant for the treatment of pain patients? Pain Manag Nurs. 2013;14(3):e67–83. 10.1016/j.pmn.2011.04.002 23972873

[28] Crown ED, Gwak YS, Ye Z, Yu Tan H, Johnson KM, Xu GY, et al. Calcium/calmodulin dependent kinase II contributes to persistent central neuropathic pain following spinal cord injury. Pain. 2012;153(3):710–21. 10.1016/j.pain.2011.12.013 PMC3367863 22296735

[29] Wang XT, Lian X, Xu YM, Suo ZW, Yang X, Hu XD. α(2) noradrenergic receptor suppressed CaMKII signaling in spinal dorsal horn of mice with inflammatory pain. Eur J Pharmacol. 2014;724:16–23. 10.1016/j.ejphar.2013.12.026 24374198

[30] Qi F, Liu T, Zhang X, Gao X, Li Z, Chen L, et al. Ketamine reduces remifentanil-induced postoperative hyperalgesia mediated by CaMKII-NMDAR in the primary somatosensory cerebral cortex region in mice. Neuropharmacology. 2020;162:107783. 10.1016/j.neuropharm.2019.107783 31541650

[31] Colvin LA, Bull F, Hales TG. Perioperative opioid analgesia-when is enough too much? A review of opioid-induced tolerance and hyperalgesia. Lancet. 2019;393(10180):1558–68. 10.1016/S0140-6736(19)30430-1 30983591

[32] Liu F, Patterson TA, Sadovova N, Zhang X, Liu S, Zou X, et al. Ketamine-induced neuronal damage and altered N-methyl-d-aspartate receptor function in rat primary forebrain culture. Toxicol Sci. 2013;131(2):548–57. 10.1093/toxsci/kfs296 PMC3551423 23065140

[33] Li J, Wu H, Xue G, Wang P, Hou Y. 17β-Oestradiol protects primary-cultured rat cortical neurons from ketamine-induced apoptosis by activating PI3K/Akt/Bcl-2 signalling. Basic Clin Pharmacol Toxicol. 2013;113(6):411–8. 10.1111/bcpt.12124 23981522

[34] Li Y, Wu ZY, Zheng WC, Wang JX, Yue-Xin, Song RX, et al. Esketamine alleviates postoperative cognitive decline via stimulator of interferon genes/TANK-binding kinase 1 signaling pathway in aged rats. Brain Res Bull. 2022;187:169–80. 10.1016/j.brainresbull.2022.07.004 35839904

[35] TTang Y, Liu Y, Zhou H, Lu H, Zhang Y, Hua J, et al. Esketamine is neuroprotective against traumatic brain injury through its modulation of autophagy and oxidative stress via AMPK/mTOR-dependent TFEB nuclear translocation. Exp Neurol. 2023;366:114436. 10.1016/j.expneurol.2023.114436 37187276

[36] Naldan ME, Taghizadehghalehjoughi A. Remifentanil reduces glutamate toxicity in rat olfactory bulb neurons in culture. Braz J Anesthesiol. 2021;71(4):402–7. 10.1016/j.bjane.2021.04.003 PMC9373102 33895216

[37] Chen CR, Bi HL, Li X, Li ZM. Remifentanil protects neurological function of rats with cerebral ischemia-reperfusion injury via NR2B/CaMKIIα signaling pathway. J Biol Regul Homeost Agents. 2020;34(5):1647–56. 10.23812/20-169-A 33103411

